# PROMOTING HEALTHY AGING THROUGH LGBTQ+ AFFIRMING CARE

**DOI:** 10.1093/geroni/igac059.169

**Published:** 2022-12-20

**Authors:** Harry Barbee, Tara McKay, Nathaniel Tran, Judy Min

**Affiliations:** Vanderbilt University, Nashville, Tennessee, United States; Vanderbilt University, Nashville, Tennessee, United States; Vanderbilt University, Nashville, Tennessee, United States; Vanderbilt University, Nashville, Tennessee, United States

## Abstract

This study examines variation in health service utilization and aging outcomes among older LGBTQ+ adults by access to an LGBTQ+ affirming health care provider. Primary survey data (n=1128) come from the Vanderbilt University Social Networks, Aging, and Policy Study, a panel study of older LGBTQ+ adults residing Alabama, Georgia, North Carolina, and Tennessee. Respondents with an LGBTQ+ affirming health care provider were more likely to seek preventative care, including routine checkups, colorectal screenings, flu shots, and HIV tests. Respondents with an LGBTQ+ affirming health care provider also reported higher control over their mental health and lower levels of cognitive decline. Overall, this study suggests that increasing access to LGBTQ+ affirming care could reduce health disparities among aging LGBTQ+ populations.

